# A new insight into the different approaches for the ablation of para-Hisian accessory pathways: safety, effectiveness, and mechanism

**DOI:** 10.1007/s10840-022-01343-5

**Published:** 2022-08-16

**Authors:** Yang Pang, Qingxing Chen, Ye Xu, Kuan Cheng, Yunlong Ling, Junbo Ge, Wenqing Zhu

**Affiliations:** Department of Cardiology, Shanghai Institute of Cardiovascular Diseases, Zhongshan Hospital, Fudan University, No.180, Feng-Lin Road, Shanghai, People’s Republic of China 200032

**Keywords:** Ablation, Para-Hisian AP, Non-coronary cusp

## Abstract

**Background:**

To compare the safety, effectiveness, electrophysiological characteristics, and mechanisms of different approaches for the ablation of para-Hisian accessory pathways (APs).

**Method:**

Eighteen consecutive patients with para-Hisian APs were enrolled in this study. Detailed mapping of retrograde conduction as well as antegrade conduction (if possible) in both the right sided His bundle region and non-coronary cusp (NCC) region was performed before ablation. Ten patients underwent initial ablation in the right septal (RS) region while the remaining 8 patients were ablated in NCC region. Repeat ablation was attempted in an alternative region if ablation at the first site failed.

**Results:**

Among the patients whose procedures were successful, 7 cases were successfully ablated with a NCC approach while 10 were conventionally ablated in RS region. For successful procedures targeting the NCC region, the earliest atrial activation (EAA) in NCC region preceded that at RS region by 4–13 ms. The distance between NCC targets and near-field His potential (NFH) points was longer than that between RS targets and NFH points. Additionally, the risk of complication after ablation in NCC region was lower compared with that following RS-targeted procedure.

**Conclusion:**

NCC approach provided a high success rate and low risk of complication for the ablation of para-Hisian APs as long as EAA was observed in NCC region. Sites of successful para-Hisian AP ablation in NCC region had different retrograde mapping patterns in comparison with successful ablation sites in the RS region.

Radiofrequency catheter ablation (RFCA) has been proved to be highly effective for the treatment of accessory pathways (APs). However, the ablation of para-Hisian APs still remains a challenge due to their special anatomic proximity to the conduction system. A higher recurrence rate and risk of atrioventricular (AV) block after procedure were also associated with patients with para-Hisian APs compared with those with conventional APs [1–3]. Previously different approaches for ablation of para-Hisian APs had been developed in order to enhance success rates as well as reduce complication rates. While an ablation in the right septal (RS) region via the inferior vena cava approach (IVC-A) was mostly utilized for para-Hisian APs, in some cases the noncoronary cusp (NCC) approach or an alternative cryoablation catheter was necessary because of unavoidable junctional rhythm or ineffective ablation attempts with the conventional method. The safety and effectiveness of NCC approach for the ablation of para-Hisian APs had been discussed by several studies [4–7]. However, it is still controversial whether we should use NCC approach for the initial ablation attempt and under what condition this approach is appropriate. In this study, the mapping and ablation characteristics and the effectiveness of NCC approach in different strategies for para-Hisian AP ablation were evaluated.

## Method

### Study population

A total of 415 patients with APs received RFCA in our center during 2019–2020. Among these patients, 18 consecutive patients with para-Hisian APs were enrolled in this retrospective study based on the following inclusion criteria. All patients enrolled in the study reported symptoms of palpitations. The documented ECGs demonstrated supraventricular tachycardia and manifest preexcitation in 17 and 8 patients, respectively. In addition, eligible patients had structurally normal hearts in echocardiographic examination and stopped using antiarrhythmic drugs for at least 5 half-lives before the procedure. The study was approved by the ethics committee of Zhongshan Hospital, Fudan University.

### Electrophysiological study

Standard electrodes were placed in the high right atrium, the coronary sinus, the His bundle (HB), and the right ventricular apex. Both bipolar and unipolar electrograms were recorded by a Prucka system (GE Healthcare, Milwaukee, WI, USA) (filtered at 30–500 Hz and 0.05–500 Hz, respectively). Standard EP study was then performed in each patient. According to the previously described criteria [8, 9], the differential diagnosis of various types of supraventricular tachycardias (SVTs) was made. Once the diagnosis of AP was confirmed, the location of the AP was further identified using regular mapping electrode catheters as well as a steerable ablation catheter. A para-Hisian AP was confirmed when a discernible HB potential was recorded (either the largest recordable HB value on an electrogram or an HB potential of > 0.1 mV) at the site of earliest atrial activation (EAA) during retrograde AP conduction or after ablation of a manifest AP, which resulted in the disappearance of ventricular preexcitation [10].

### Mapping and ablation

A 7-Fr saline-irrigated tip ablation catheter with a 3.5-mm distal electrode and 2–5-2 mm interelectrode spacing was used for mapping and ablation. First, three-dimensional electroanatomic mapping (CARTO, Biosense Webster, Diamond Bar, CA) was performed in all patients. Once a diagnosis of para-Hisian APs was confirmed, the conduction characteristics of APs in the right septal (RS) region as well as that in the NCC region were both carefully evaluated. The sites with earliest atrial activation during retrograde conduction mapping in both regions were defined as best targets (BTs) under most circumstances. If SVTs could not be induced, then the sites with earliest ventricular activation during antegrade conduction mapping (with manifest preexitation) were considered as BTs. AP potential in the regions of BTs was carefully evaluated in order to obtain better BTs for ablation attempt. In situations where AP potential could not be recorded, ablation was performed targeting the BT site. The BTs in both regions were marked and the distance between these two targets was calculated for each patient through the Carto system.

Two different ablation strategies were adopted in this study. For the first 10 patients (RS group), the initial ablation attempt was performed in the BTs of RS region. If the first several attempts failed or unavoidable junctional rhythm and AV block were observed during the procedure, then the operator would shift to target the NCC region. Likewise, for the remaining 8 patients (Non-Coronary Cusp Group) in whom NCC region was the target of initial attempts, the operator would choose RS region in the case of failure. The success rate and complication rate of ablation for procedures performed in both regions were compared.

Ablation energy applied in para-his region in RS was initiated at 10–15 W and titrated up to 20–40 W for a maximum temperature of 55 °C. If the best target was located just in the region with the highest local (near-field) His potential, the ablation would be performed in the near region with or without a far field His potential in order to avoid AV conduction block. Ablation in NCC region was initiated at 30 W and titrated up to 35 W.

### Patients’ follow-up

All patients received ECG monitoring after the procedure until discharge from the hospital and were followed up through visits at the cardiology outpatient department thereafter. Clinic ECGs and 24-h Holter monitoring were performed 1 month, 3 months, and 6 months after the procedure.

### Statistical analysis

Results were expressed as mean ± standard deviation (SD). Statistical differences were evaluated by *t*-test for normally distributed data or by Wilcoxon test for data following a skewed distribution. All analysis was run on the SPSS and a *p* value < 0.05 was considered statistically significant.

## Results

### Electrophysiological characteristics

The baseline characteristics of patients are shown in Table 1. Preexcitation was present in 44.4% (8/18) patients. Sustained orthodromic atrioventricular reentrant tachycardia (AVRT) was induced in 94.4% (17/18) patients. The average cycle length for patients with AVRT was 320 ± 22.9 ms. The details about the excitation mapping of antegrade and retrograde conduction characteristics for APs can be found in Table 1. A better BT of antegrade conduction mapping was found in the RS region in 4 patients while similar BTs (difference ≤ 3 ms) between two regions of antegrade mapping were observed in the remaining 4 patients. The EAA target of retrograde conduction mapping was found in the NCC region of 7 patients and in the RS region of 10 patients. In the remaining 1 patient, similar BTs were observed between the RS and NCC regions.

**Table 1:** Detailed mapping and ablation results of 18 patients with para-Hisian APs

	Sex	Age	Arrhythmia	Initial target	Final target	D(N–H) (mm)	D(R-H) (mm)	D(N-R) (mm)	Retro-mapping (N-R) (ms)	Antero-mapping (N-R) (ms)	Complication (long term)	Results	
												NCC	RS
1	M	22	CBT + AVRT	RAS	NCC	5	13.5	10.1	13	/	N	S	F
2	M	20	CBT + AVRT	RMS	RMS	12.1	8.8	9.6	− 8	/	N	/	S
3	M	37	CBT + AVRT	RAS	RAS	11.3	7.5	9.8	− 5	/	N	/	S
4	M	24	WPW + AVRT	RAS	NCC	10.1	7.1	5.2	5	3	N	S	F
5	M	44	CBT + AVRT	RMS	NCC	11.1	5.4	12.7	8	/	N	S	F
6	M	48	WPW + AVRT	RAS	RAS-V	10.5	5.2	10.3	− 5	− 3	N	F	S
7	M	42	WPW + AVRT	RAS	RAS-V	10.9	6.3	11.1	− 9	− 10	N	F	S
8	M	33	CBT + AVRT	RAS	RAS	11.3	4.7	10.5	− 11	/	N	F	S
9	F	27	CBT + AVRT	RAS	NCC	10.2	4	9.4	10	/	N	S	F
10	M	20	WPW + AVRT	RAS	Fail	8.8	3.1	9.7	− 8	− 20	N	F	F
11	M	30	CBT + AVRT	NCC	RAS	13	11.2	8.5	− 10	/	N	F	S
12	M	18	WPW + AVRT	NCC	NCC	6.0	4.0	3.5	5	− 19	N	S	/
13	F	24	WPW	NCC	RMS	8	7.7	3.2	− 8	0	N	F	S
14	M	26	WPW + AVRT	NCC	RMS	10	8.8	14.4	− 20	− 25	N	F	S
15	M	59	CBT + AVRT	NCC	RAS	7	11.5	15.6	− 21	/	N	F	S
16	M	31	WPW + AVRT	NCC	RAS	5.3	4.3	9.1	− 1	0	N	F	S
17	M	40	CBT + AVRT	NCC	NCC	9.2	8.2	7.8	8	/	N	S	/
18	F	27	CBT + AVRT	NCC	NCC	8.2	6.8	7.2	4	/	N	S	/

*F*, failed; *S*, succeed; *N*, none; *CBT*, concealed bypass tract; *AVRT*, atrioventricular reentrant tachycardia; *WPW*, Wolf-parkinson-white syndrome; *RS*, right septal; *RAS*, right anterior septal; *RMS*, right medium septal; *NCC*, noncoronary cusp; *RAS-V*, ventricular aspect of right anterior septal (with a reverse-U technique of catheter); *D(N–H)*, The distance between the NCC targets and the near-field His potential points; *D(R-H)*, the distance between the right septal targets and the near-field His potential points; *D(N-R)*, the distance between the best targets of right septal and NCC region; *Retro-mapping(N-R)*: the difference of the preceding time of retrograde conduction mapping between the best targets of NCC and RS region. A positive value indicated an earliest atrial retrograde activation in NCC region; *Antero-mapping(N-R)*, the difference of the preceding time of antegrade conduction mapping between the best targets of NCC and RS region. A positive value indicated an earliest ventricular antegrade activation in NCC region

### Catheter ablation

**Fig. 1:**
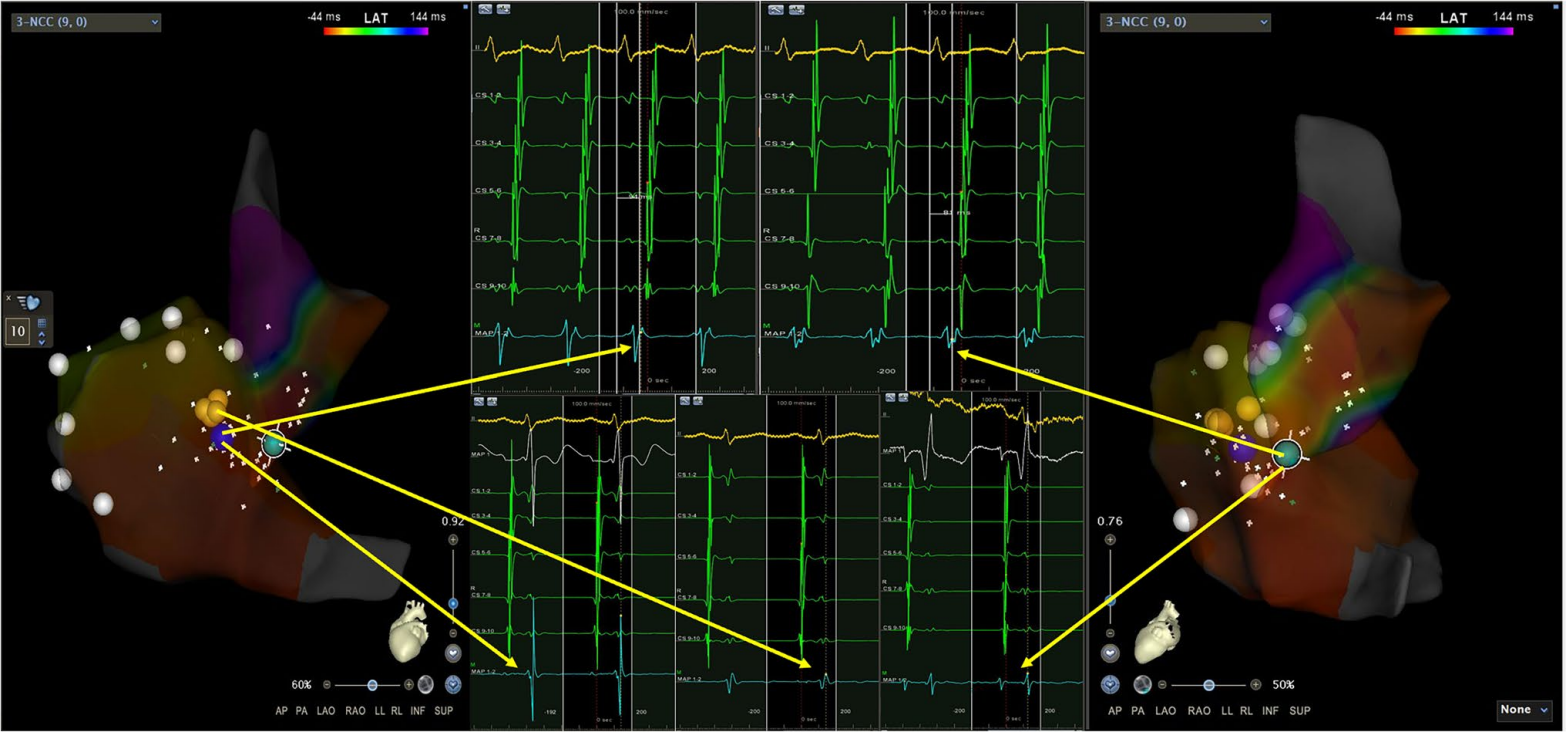
Mapping and ablation of para-Hisian APs with targets in NCC region: BTs in NCC and RS region were showed as green point and blue point respectively. The unipolar and bipolar electrograms of BTs were illustrated in the figures. A best target (green point) in retrograde conduction mapping of APs during tachycardia was found in NCC region. This point preceded the best target (blue point) in right septal region by 13 ms. The point with His potential was illustrated as yellow point. A first ablation attempt was performed in the right septal region (blue point) but failed. The APs were successfully ablated in the NCC region (green point) finally

**Fig. 2:**
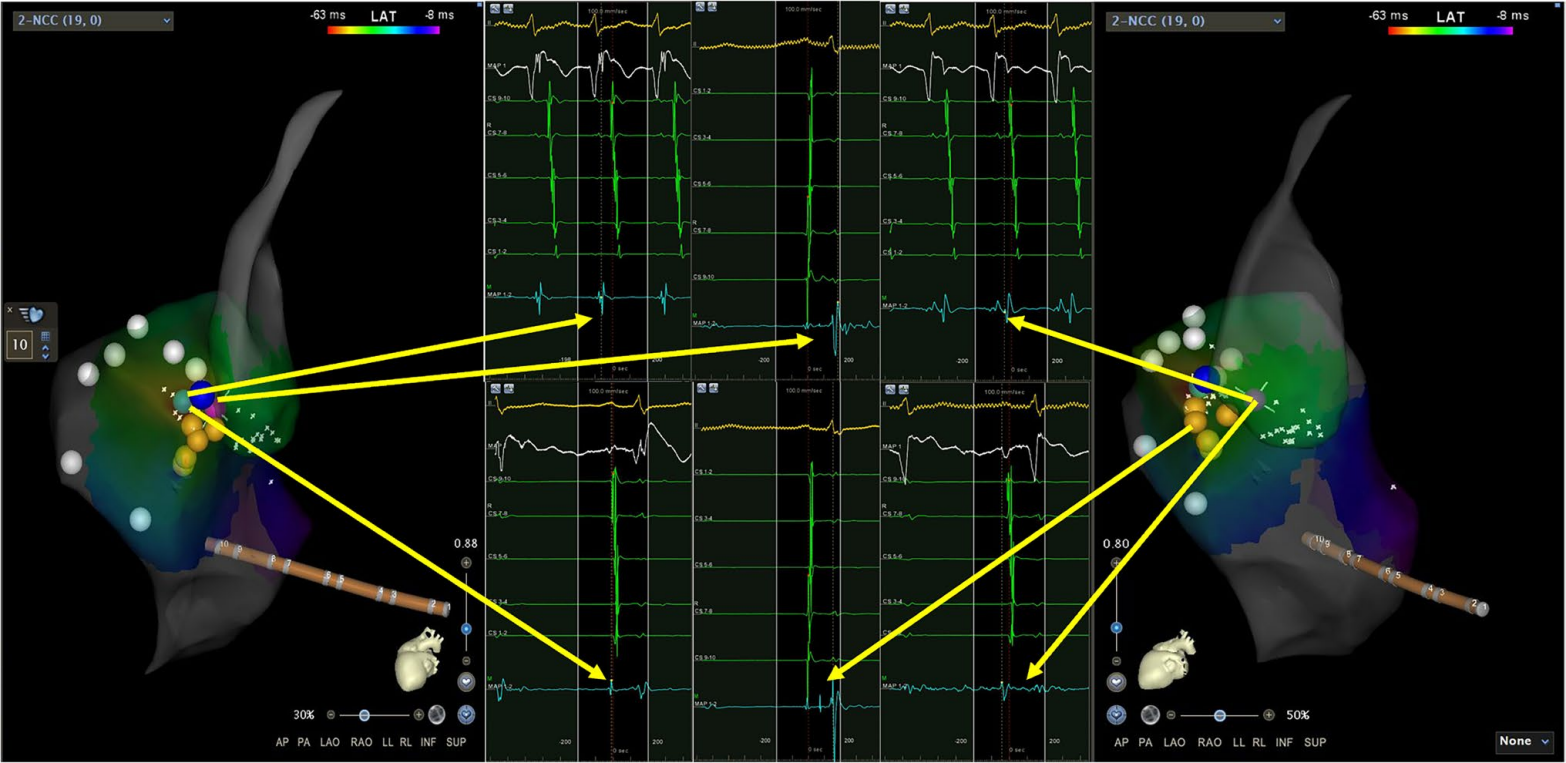
Mapping and ablation of para-Hisian APs with targets in right septal region: BTs in NCC and RS region were showed as purple point and green point respectively. The unipolar and bipolar electrograms of BTs were illustrated in the figures. A best target (green point) in retrograde conduction mapping of APs during tachycardia was found in right septal region. The earliest atrial retrograde activation site (purple point) in LVOT located in the NCC region adjacent to the right coronary cusp. The retrograde atrial activation of green point preceded that of purple point in NCC region by 21 ms. A far field His potential was recorded near this target during sinus rhythm (blue point). A biggest near-field His potential (orange point) was recorded 11.5 mm under this target. A first ablation attempt was performed in the NCC region (purple point) but failed. The APs was successfully ablated in the right septal region (green point) finally

Ablation of targeted APs was achieved in 17 patients. Among them, 7 patients were successfully ablated using a NCC approach, while 8 patients were successfully ablated in the RS region, and 2 patients on the ventricular aspect of tricuspid annulus with a reverse-U technique. The AP in the remaining patient could only transiently suppressed in the RS region. In this patient, ablation attempts were stopped due to rapid junctional rhythm as well as first degree AV block which lasted for 10 s even with a power of 10 W. AP function recovered immediately after we stopped ablation. Due to high risk of complications and ineffective attempts in the NCC region, the decision was made to terminate further ablation in this patient. This patient received a repeat ablation in the RS region with a cryoablation catheter 6 months later and was successful. In RS group, 5 patients succeeded in the RS region while 4 patients succeeded in the NCC region. For the 4 patients failed in the initial attempts in the RS region, transient suppression of AP conduction as well as unavoidable rapid junctional rhythm were observed during ablation. However, the function of APs recovered immediately once the ablation was discontinued. In the Non-Coronary Cusp Group, 5 patients succeeded in the RS region while 3 patients succeeded in the NCC region. Transient suppression of AP conduction was observed in 2 patients who failed in the initial attempts in the NCC region. In patients who succeeded in the NCC region, all the EAA targets were located in the NCC region. The final BT targets preceded those in the RS region by 7.6 ± 3.2 ms (Fig. 1). In patients who obtained success in the RS region, two retrograde conduction patterns were observed. The BTs in the RS region preceded those in the NCC region by 5–21 ms in 9 patients (Fig. 2), while similar BTs between two regions were observed in the remaining 1 patient. In this study, AP potential were recorded in 11.7% (2/17) patients from NCC region and in 41.2% (7/17) patients from RS region during a retrograde mapping. The distances between the BTs of RS and NCC regions (D_R-N_) were 9.3 ± 3.2 mm. The distances between the NCC targets and the NFH points (D_N-H_) were longer than those between the RS targets and the NFH points (D_R-H_) (9.3 ± 2.3 mm vs 7.1 ± 2.9 mm, *p* = 0.03). The details of ablation targets are illustrated in Table 2. A positive N-R value (> 3 ms) had a high predictive value for successful ablation of a para-Hisian accessory pathway from the non-coronary cusp with a sensitivity of 100% and a specificity of 100%.

**Table 2:** The electrophysiological characteristics of ablation targets of NCC and RS region

Target	NCC	RS	*P* value
Ablation power	30–35 W	15–40 W	/
Complication (Temporarily)			
Rapid junctional rhythm	0/16	8/15	< 0.01
AV block (total)	0/16	3/15	0.10
I°AVB	0/16	3/15	0.10
II°AVB or higher	0/16	0/15	> 0.05
Distance to His (mm)	9.3 ± 2.3	7.1 ± 2.9	0.03
Success rate	7/16	10/15	> 0.05
Potential characteristic			
Near-field His potential	0/16	0/15	> 0.05
Far-field His potential	0/16	12/15	< 0.01
Without His potential	16/16	3/15	< 0.01

*RS*, right septal; *NCC*, noncoronary cusp

### Complications and follow-up

Following ablation in the RS region, complications including temporarily rapid junctional rhythm (in 53.3% (8/15) patients) and transient first-degree AV block (in 20% (3/15) patients) were observed. However, AV conduction in these three patients recovered several minutes after ablation was stopped. None of these complications occurred when ablation was performed in the NCC region.

During a mean follow-up period of 19.5 ± 4.5 months, 94.4% (17/18) patients were free of arrhythmias without anti-arrhythmic drugs. In the remaining one patient whose AP was successfully ablated in the RS region with a maximum power of 25 W, a documented ECG taken 1 month after procedure indicated manifest preexcitation. This patient refused to receive a second ablation procedure.

## Discussion

### Major findings

The main findings of this study were as follows: (1) A relatively high percent of para-his APs could be safely and effectively ablated in the NCC region. (2) Compared with conventional ablation targeting the RS region, the NCC approach resulted in a lower rate of complication. (3) Para-Hisian APs which were ablated successfully in the NCC region had different retrograde mapping patterns from those ablated in the RS region. Hence, the initial ablation attempt was recommended in the NCC region when EAA points of retrograde mapping were observed in that region.

### The characteristic of mapping and ablation

In this study, the multiple patterns of retrograde AP conduction mapping yielded different ablation results. In 7 patients whose APs were ablated in the NCC region, the EAA in that region preceded that at RS region by 4–13 ms. In 10 patients whose final targets were located in the RS region, two separate mapping results were observed: The EAA in the RS region preceded that at the NCC region in 9 patients by 5–21 ms, while similar preceded targets between the RS and NCC regions were observed in the remaining 1 patient. This result indicated that the retrograde conduction map might have a predictive value in choosing an appropriate ablation target. Specifically, NCC approach was preferred as the initial ablation attempt when earlier atrial activation in retrograde mapping was found in the NCC region. Otherwise, the conventional approach should be used instead. In this study, AP potential was recorded in 11.7% (2/17) patients from the NCC region and in 41.2% (7/17) patients from the RS region during a retrograde mapping. Though the presence of AP potential was used to define appropriate ablation sites, there was likely heterogeneity between operators in determining presence of AP potentials during review of electrograms at BT sites. The validation of a signal as a true AP potential is a tedious process. The presence of a presumed AP potential is reported at only 37–67% of successful ablation sites during retrograde AP conduction mapping [11–13]. That is why we chose the sites with EAA as BTs for ablation attempts in this study.

### The mechanism of the NCC approach for para-Hisian AP ablation

The anatomic relationship between NCC and RS targets was also further explored in this study through catheter mapping. There were longer distances between NCC targets and NFH points than those between RS targets and NFH points. The distance between the BTs located amid the NCC and RS regions was 9.3 ± 3.2 mm. Although those distances in some patients were no more than 5 mm, an ablation attempt in the NCC region failed to affect the function of APs, which necessitated the switch to the RS region to achieve success. For some patients with a N-R distance more than 10 mm, an ablation in NCC region could temporarily influence the AP function while the final targets were located in the relevant RS region. Previous studies had documented the relationship between NCC and AV conduction system [14]. The junction of the His bundle with the left bundle branch was found just below the aortic valve and in the region between the right coronary cusp and NCC. The compact AV node was located approximately 5–10 mm below the base of the NCC on the right side of the interatrial septum. To some extent, NCC was anatomically closer with the left-sided His bundle than the right-sided region. Moreover, previous study [15] had proved that RF applications in the NCC region resulted in transmural left atrial lesions which were consistently visualized on the septal atrial wall between the mitral annulus and the inferior border of the fossa ovalis. Therefore, the NCC can be an alternative target for catheter ablation of the para-Hisian APs if there was failed previous ablation in the RS. Previously the mechanism of a successful ablation in the NCC region for the treatment of para-Hisian AP was thought to be an anatomical ablation rather than a mapping ablation. However, in our study, the para-Hisian APs ablated successfully in the NCC region had a totally different retrograde mapping result from those succeeded in the RS region, a result consistent with previous studies [4, 10]. There was no correlation between the outcome of ablation and the N-R distance. Therefore, the anatomic location might vary between the para-Hisian APs which were ablated successfully in different regions, namely the NCC region and the RS region. It was supposed that the para-Hisian APs could be further divided into two types: (1) left-sided para-Hisian APs: the atrial insertion site of APs was located on the left side of interatrial septal, which resulted in a retrograde EAA in the NCC region; (2) right-sided para-Hisian APs: APs were located in the typical right atrium to HB region which could be eliminated in the RAS region.

### Different ablation approaches for para-his APs

The ablation of para-Hisian APs had long been a clinical challenge with its high risk of AV node damage as well as high rate of recurrence. It was unavoidable of a rapid junctional rhythm or AV damage for some patients during ablation performed in the right-sided HB region. Although the cryoablation technique could decrease the risk of AV damage, the long-term recurrence rate was still highly debated in previous studies [16]. In previous studies [7, 17], NCC approach was recommended as an alternative to a failed attempt in the RAS region. However, according to our study, a detailed retrograde mapping in the NCC before the first ablation had unique value in predicting the outcome of procedure. Earliest BT site in the NCC was led to high success rate and low risk of complication with first ablation attempts in this region. Otherwise, the right-sided HB region should be selected as the target of the initial ablation attempt either with a conventional method or with a reverse-U technique, given that a NCC approach might prove to be ineffective for these patients. Ablation using a cryocatheter should be considered if risk of serious complications in the RAS region are high.

### Limitation

The present study had several limitations. (1) The retrospective design of the study is associated with risks of bias in comparing different ablation approaches. Randomized controlled trials or prospective studies are needed to confirm our conclusion. (2) In our study, the ablation strategy was determined by the detailed retrograde mapping results of right HB and NCC region. However, there were no generally accepted criteria as to the definition of similar BTs of these two regions. (3) Given the low incidence of para-Hisian APs, the small sample size is prone to bias the results of this study and requires further assessment in larger cohorts.

## Data Availability

The data that support the findings of this study are available from the corresponding author on reasonable request.
